# Gender Differences in Associations between Biomechanical and Psychosocial Work Exposures and Age of Withdrawal from Paid Employment among Older Workers

**DOI:** 10.3390/ijerph191710563

**Published:** 2022-08-24

**Authors:** Karina Undem, Taina Leinonen, Petter Kristensen, Suzanne L. Merkus, Rachel L. Hasting, Jon Michael Gran, Ingrid S. Mehlum

**Affiliations:** 1 National Institute of Occupational Health, 0363 Oslo, Norway; 2 Finnish Institute of Occupational Health, 00250 Helsinki, Finland; 3 Oslo Centre for Biostatistics and Epidemiology, Department of Biostatistics, Institute of Basal Medical Sciences, University of Oslo, 0372 Oslo, Norway; 4 Institute of Health and Society, University of Oslo, 0450 Oslo, Norway

**Keywords:** biomechanical exposure, gender, gender differences, older workers, psychosocial exposure, retirement age, withdrawal from work, work exposure

## Abstract

Background: Work exposures are known predictors of withdrawal from employment, but the associations between work exposures and withdrawal may vary with gender. This study evaluated gender differences in associations between biomechanical and psychosocial work exposures and age of withdrawal from paid employment among older workers in Norway. Methods: 77,558 men and 67,773 women (born 1949–1953) were followed from age 62 until withdrawal from paid employment or end of follow-up in 2016 (up to five years follow-up). Information about eight biomechanical and seven psychosocial exposures was obtained from a gender-specific job exposure matrix. Using Cox regression, the difference in mean estimated time until withdrawal between non-exposed and exposed was calculated for each gender and work exposure separately. Results: The largest gender difference was found for high psychological demands. Among men, the non-exposed withdrew earlier than the exposed (−3.66 months (95% CI: −4.04–−3.25 months)), and contrary among women (0.71 (0.28–1.10)), resulting in a gender difference of 4.37 (3.81–4.97) months. Gender differences were also found for monotonous work (4.12 (3.51–4.69) months), hands above shoulder height (2.41 (1.76–3.10) months), and high iso-strain (2.14 (1.38–2.95) months). Conclusions: There were observed gender differences in the associations between some biomechanical and psychosocial work exposures and mean age of withdrawal from paid employment among older workers. However, the results are likely affected by the selection of who remains in the workforce at age 62 and should be interpreted accordingly.

## 1. Introduction

With an ageing population, the number of retired in relation to working individuals increases, which constitutes a significant challenge to public finances. In order to secure the financial basis of a welfare state, it is important to maintain high work participation among older workers [1]. Several countries, including Norway, have implemented reforms to prevent early withdrawal from work [2]. However, withdrawal from work is a complex process, with multiple interacting individual, workplace and societal factors influencing the length of working life [3].

Working conditions, such as adverse psychosocial and biomechanical work exposures, can contribute to pushing the worker out of work [4]. For instance, harmful work exposures could affect the motivation to work [5], or there may be a discrepancy between work requirements and work ability, either directly or through health effects, making it challenging or impossible to perform the work [6]. On the other hand, work exposures may also contribute to prolonging working life [4], for example by facilitating skill development and skill utilisation [7]. Thus, knowledge of how work exposures are associated with early withdrawal from work among older workers is vital to extend working life successfully. Previous studies have examined biomechanical and psychosocial work exposures among older workers with respect to early retirement [8,9,10,11,12,13,14,15,16,17,18], disability retirement [8,14,16,18,19,20], long-term sickness absence [16,19] and unemployment [8,16,19]. 

However, men and women may have different push and pull factors [21], and there is a lack of studies focusing on gender differences in the relationship between work exposures and withdrawal from work among older workers. First, only a few studies have reported gender-specific results [14,18,20], although some report that stratified or interaction analysis did not reveal substantial gender differences [13,19]. Second, among the studies that reported gender-specific results, the findings have been inconsistent for psychosocial exposures. High job demands have been found to be a risk factor for disability pension and early retirement pension among women, but both a risk factor and a protective factor among men [14,18]. Inconsistent results with regard to disability pension and early retirement have also been found for low job control, with a mix of positive, negative and no association for men and women [14,18,20]. Third, there is a lack of studies examining multiple independent work exposures, particularly among biomechanical exposures. Fourth, most commonly, only one or two work exit routes have been examined at a time, usually they are exit through disability pension or retirement pension. However, work exposures may impact each exit route differently, and men and women tend to take different routes. For instance, studies from Norway have shown that women are more prone to receive disability pension [22], whilst men are more likely to withdraw early retirement pension [23]. Therefore, simultaneously examining multiple routes out of work will provide better insight into any potential gender difference in the relationship with work exposures. 

A comprehensive understanding of whether older men and women have different work-related predictors of early withdrawal from work is currently lacking. Knowledge of such gender differences is important to successfully implement interventions aimed at extending age of withdrawal from work. If differences between genders exist, policymakers and employers should consider gender-targeted interventions to extend working life among older workers. 

The present study aimed to examine gender differences in associations between biomechanical and psychosocial work exposures at age 62 and age of withdrawal from paid employment. When interpreting the associations, it should be noted that the population of employed individuals at age 62 is selected, for instance, due to previous work exposure and health. 

## 2. Materials and Methods

### 2.1. Data Sources, Study Population and Study Design

The data come from a registry-based cohort of all working-age individuals who have resided in Norway between 2000 and 2010 (complete cohort). Register data until 2016 were retrieved for all individuals from various nationwide registries and linked by means of the unique national identification number. Longitudinal individual data on employment (unreported employment, such as illicit work, is not recorded, and therefore not included), occupation, working hours, pensions and other welfare benefits, level of education, civil status, residential status, age and gender were obtained from Statistics Norway [24]. Monthly data were available for all variables, except for working hours and employment after June 2014 for which annual data was utilised.

**Figure 1:**
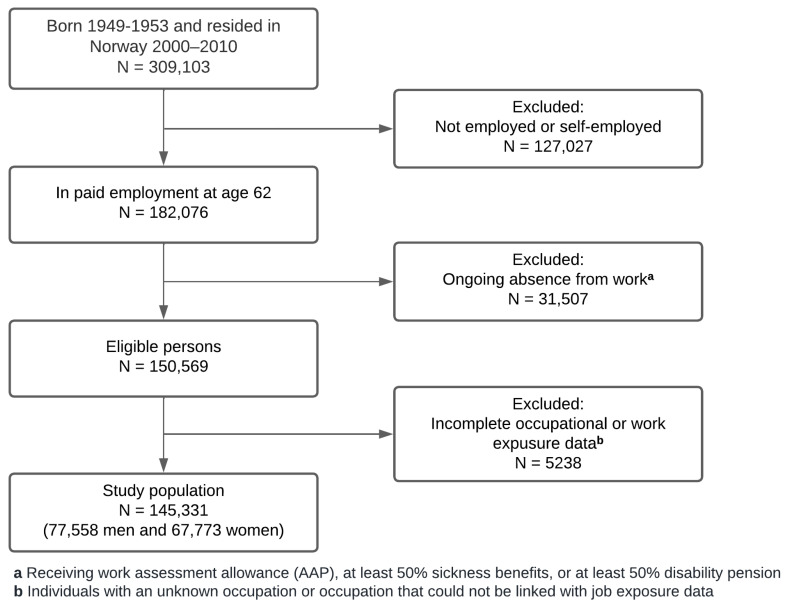
Flow diagram of the study selection process.

The dynamic study population included in the present study consisted of all individuals born between 1949 and 1953 who were in paid employment, either when turning 62 (if born 1949–June 1952), or in November prior to turning 62 (if born after June 1952, for whom monthly employment information was lacking) (N = 182,076; see Figure 1). Individuals with ongoing absence from work, i.e., receiving at least 50% sickness benefits, work assessment allowance (AAP; usually after having received sickness benefits for the maximum of 12 months), or at least 50% disability pension, when turning 62, were excluded (17%). Furthermore, individuals with an unknown occupation (3%) or an occupation that could not be linked with a previously developed job-exposure matrix (JEM) (<1%) [25] were excluded. During the follow-up period of 2011–2016, study participants were included the month they turned 62 and followed up until either withdrawal from paid employment, age 67 (the historical statutory retirement age), or end of follow-up in June 2016. 

### 2.2. Withdrawal from Paid Employment

The process of transitioning out of paid employment is complex with multiple possible work exit routes for older workers [26]. The main route out of paid employment in Norway is through old age retirement [2]. In 2011, a flexible retirement age arrangement was implemented. Given sufficient pension savings, workers can retire part-time or full-time from the age of 62 on retirement pension (contractual early retirement pension (AFP) and/or state retirement pension). The retirement pension can be combined with part-time or full-time work. The present study started follow-up in 2011 to ensure that all participants followed the post-reform retirement pension system. Another route for withdrawal from paid employment is through health-related benefits and pensions, i.e., long-term sickness absence (benefits can be received for 52 weeks), AAP (up to four years at the time of the study) or disability pension (until age 67). A third route is to withdraw from paid employment due to long-term unemployment (benefits can be received for up to two years). 

Depending on pension savings and health status, not all workers are eligible for all routes of withdrawal. Some workers are eligible for more than one route and may, for instance, choose to withdraw on retirement pension instead of disability pension [27]. Thus, to take into account the complexity of competing exit routes, the main study outcome was age at *all-cause* withdrawal from paid employment. A secondary study outcome was *cause-specific* withdrawal from paid employment. Furthermore, because work and retirement are commonly combined, the definition of withdrawal from paid employment incorporated both complete and gradual withdrawal from paid employment. The lower withdrawal limit was set to 50% to capture the transition from primarily employed to primarily withdrawn from paid employment. 

All-cause withdrawal from paid employment was defined as the first episode of one of the following events:

i. Retirement: receiving at least 50% flexible retirement pension and working fewer than 20 h per week.
ii. Withdrawal due to health-related reasons: (a) receiving at least 50% disability pension, or (b) receiving at least 50% sickness benefits or AAP for at least six months, or fewer than six months if followed by a transition to disability pension.
iii. Unemployment: registered as fully unemployed for at least six months.
iv. Death or emigration.
v. Other reasons: registered as not employed two years in a row in the annual labour market records.

Cause-specific withdrawal from paid employment was defined as a first episode of one of the specific events (i)–(v). Event (v) consists of people who, among other reasons, transferred to self-employment or received a special occupational pension from their employer.

### 2.3. Work Exposures

Information about work exposures was obtained by linking a gender-specific JEM to occupation at baseline (occupation when turning 62), coded according to the Norwegian version of the International Standard Classification of Occupations (ISCO-88) [28]. The JEM is based on the Norwegian nationwide Survey of Living Conditions on work environment in 2006 and 2009 [25]. Self-reported work exposures from 18,939 individuals aged 18–66 were used to estimate dichotomised work exposures for 268 occupational groups at the four-digit level. For small occupational unit groups where gender-specific exposure estimates were unachievable, combined exposure estimates for men and women were calculated if experts assessed men and women to have similar work tasks and work exposures. Gender-specific exposure estimates were achieved for 132 (49%) of the 268 occupational groups.

Eight biomechanical exposures from the JEM were examined: *heavy lifting* (lifting more than 20 kg at least once during the workday), *neck flexion* (working with the head bent forward ≥ ¼ of the workday), *hands above shoulder height* (working with hands raised to the shoulder height or higher ≥ ¼ of the workday), *squatting/kneeling* (≥¼ of the workday), *forward bending* (leaning forward without supporting oneself on the hands or arms ≥ ¼ of the workday), *awkward lifting* (lifting in uncomfortable positions ≥ ¼ of the workday), *heavy physical work* (work that causes more rapid breathing ≥ ¼ of the workday) and *standing/walking* (≥½ of the workday). Four psychosocial exposures, based on 14 items, mostly from the General Nordic Questionnaire for Psychological and Social Factors at Work [29], were included from the JEM: *monotonous work* (one item: work that consists of constantly repeated tasks), *decision latitude* based on decision authority (three items: able to decide pace, influence decisions and decide how to perform the work) and skill discretion (two items: use of skills, knowledge and experience and opportunity to develop these skills), *supportive leadership* (three items: support from your immediate superior, appreciation from your immediate superior and fair and impartial treatment from your immediate superior), *psychological demands* based on quantitative demands (one item: have much work to do), role conflict (three items: receive contradictory requests, given tasks without being given sufficient tools and resources to complete them, do work one thinks should be done in a different way) and emotional demands (one item: need to deal with strong feelings such as sorrow, anger, desperation, frustration and so on from customers, clients or other people not employed at the workplace). In addition, *job strain* (combination of high psychological demands and low decision latitude), *iso-strain* (combination of high psychological demands, low decision latitude and low support from leadership) and *emotional demands* (which is one of the three components of psychological demands) were included in the present study. The psychosocial exposures variables were dichotomised by splitting the individual and the occupational group scores at the median. See Hanvold, Sterud, Kristensen and Mehlum [25] for detailed information about the creation of the exposure variables. 

### 2.4. Covariates

Civil status and level of education were measured at baseline (when turning 62). Civil status was grouped into three categories: married/cohabiting, single (including divorced, separated and widow/widower) and unknown civil status. Level of education was based on the Norwegian Standard Classification of Education (NUS2000) [30] and grouped into six categories: lower secondary level or lower (NUS 0–2), upper secondary basic level (NUS 3), upper secondary final year (NUS 4–5), bachelor level (NUS 6), master level or higher (NUS 7–8) and unknown educational level. In addition, calendar year at baseline (2011–2015) was included to account for differences between birth cohorts in withdrawal behaviour and work exposures.

### 2.5. Statistical Analysis

The association between work exposures at baseline (when turning 62) and age of withdrawal from paid employment was examined by Cox proportional hazards models, stratified by work exposure and gender. Age was used as the underlying time scale, and time was measured in months since the study participants turned 62. Study participants were censored when they turned 67 or at the end of the follow-up period, December 2016. Survival curves were graphed by modifying the estimated baseline survival function with covariates set to mean (civil status: married/cohabiting; level of education: upper secondary final year; and calendar year: 2013). Mean estimated time until withdrawal from paid employment was calculated as the area under the survival curves, that is, time spent in paid employment until withdrawal between age 62 and 67. 

The difference in mean estimated time until withdrawal between non-exposed and exposed was calculated for each work exposure:

$$\left[{\mathrm{Employment}\;\mathrm{time}}_{\mathrm{non}-\mathrm{exposed}\;}-{\;\mathrm{Employment}\;\mathrm{time}}_{\mathrm{exposed}\;}\right]$$

A positive difference indicated that non-exposed individuals withdrew from paid employment later than exposed individuals, given covariates at mean, whereas a negative difference indicated that non-exposed withdrew at a younger age than exposed individuals. We thereafter calculated the gender difference (men–women) in the difference in mean estimated time until withdrawal:

$$\left[{\mathrm{Employment}\;\mathrm{time}}_{\mathrm{non}-\mathrm{exposed}\;\mathrm{men}}-{\;\mathrm{Employment}\;\mathrm{time}}_{\mathrm{exposed}\;\mathrm{men}}\right]\;-[{\mathrm{Employment}\;\mathrm{time}}_{\mathrm{non}-\mathrm{exposed}\;\mathrm{women}}-{\;\mathrm{Employment}\;\mathrm{time}}_{\mathrm{exposed}\;\mathrm{women}}]$$

A positive gender difference illustrates that the exposure was associated with a higher risk of withdrawal among men; a negative gender difference illustrates that the risk was higher among women. Finally, 95% confidence intervals (CI) were estimated based on 1000 bootstrap samples. 

The proportional hazard assumption was assessed visually by examining the log–log hazards plots and comparing the Kaplan–Meier survival curves with the predicted survival curves. No clear violations of the proportional hazard assumption were found. 

Post hoc analyses were performed on work exposures that showed a large gender difference in their association with age of withdrawal from paid employment. Cause-specific withdrawal from paid employment due to retirement (withdrawal event (i) in Section 2.2) and health reasons (withdrawal event (ii)) were examined with cause-specific cumulative incidence curves by gender and exposure. The cumulative incidence functions were estimated at the mean values of the abovementioned covariates and by taking competing risks into account [31]. 

All statistical analyses were conducted using STATA version 16.1 (StataCorp LLC, College Station, TX, USA).

### 2.6. Sensitivity Analyses

Only 10% of the study population was followed until age 67 (the historical statutory retirement age), as most participants were 66 or younger at the end of the study’s follow-up in 2016. To examine the consequences of this, a sensitivity analysis was performed using individuals born January–June 1949 who could be followed up until age 67. To assess the accuracy of employment status at baseline, sensitivity analyses were performed by excluding individuals born after June 1952, for whom only annual, instead of monthly employment information, was available. Furthermore, to assess how the relationship between work exposures and age of withdrawal depended on the definition of retirement, the analyses were rerun with a definition of retirement solely based on retirement pension received (receiving at least 50% flexible retirement pension). In contrast to the original definition (receiving at least 50% flexible retirement pension and working fewer than 20 h per week), individuals receiving this pension and working 20 h or more per week were now classified as retired.

## 3. Results

The final study population comprised 145,331 individuals (77,558 men and 67,773 women) (Table 1). As follow-up time was shorter for younger birth cohorts, the proportion of withdrawal decreased with birth year. Among individuals not censored due to end of follow-up (born January–June 1949), 66% withdrew from paid employment before age 67; withdrawal was somewhat more common among women (69%) than men (64%). Among men, singles were slightly more likely to withdraw (48%) compared with married/cohabiting men (45%), whereas the opposite was found among women (44% and 52%, respectively). The proportion of withdrawal was higher among the low educated than highly educated for both men and women. Among men, the most frequent exposure was low supportive leadership (52%). Among women, high emotional demands were the most common (75%). Compared with men, women had a higher prevalence for most work exposures, except for heavy lifting, hands above shoulder height and heavy physical work. Retirement was the most common cause-specific withdrawal, followed by withdrawal due to health reasons, other reasons, unemployment and death/emigration (Table 2).

The results from the Cox proportional hazards models, that is, the hazard ratios (HRs) of all-cause withdrawal from paid employment and corresponding 95% CIs, can be found in Table S1. The difference between non-exposed and exposed in the probability of being in paid employment at age 62 to 67 (i.e., difference in survival curves) is graphed in Figures S1 and S2.

Table 3 shows the difference in mean time (months) until all-cause withdrawal from paid employment between non-exposed and exposed, for men and women. After taking baseline characteristics into account, time until withdrawal was longer among non-exposed than exposed for all work exposures, except for high psychological demands for men, hands above shoulder height for women and monotonous work for women. The difference in mean time until withdrawal between non-exposed and exposed ranged from 1.02 months (95% CI: 0.67–1.37 months) (low supportive leadership) to 4.43 (4.07–4.81) months (standing/walking) for men and from −0.08 (−0.62–0.47) months (hands above shoulder height) to 3.00 (2.58–3.43) months (low decision latitude) for women. In general, the difference in mean time until withdrawal between non-exposed and exposed was higher among men than women.

The gender difference (men–women) in the difference between non-exposed and exposed in mean time until withdrawal was the largest for high psychological demands. In contrast to most exposures, non-exposed men withdrew earlier than exposed men (−3.66 (−4.04–−3.25) months), with the opposite true for women (0.71 (0.28–1.10) months), resulting in a gender difference of −4.37 (−3.81–−4.97) months. Large gender differences were also found for monotonous work (4.12 (3.51–4.69) months), hands above shoulder height (2.41 (1.76–3.10) months) and high iso-strain (2.14 (1.38–2.95) months). A gender difference of fewer than one month was found for heavy lifting, neck flexion, squatting/kneeling, awkward lifting, forward bending, heavy physical work and low supportive leadership. 

**Figure 2:**
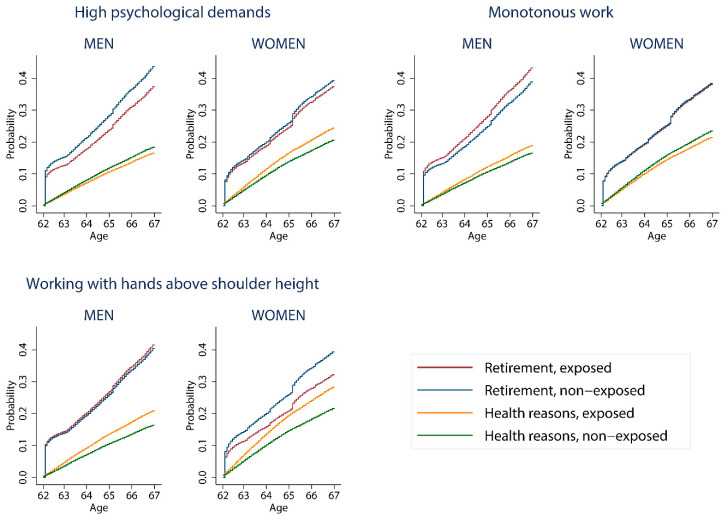
Cumulative incidence curves for cause-specific withdrawal from paid employment (retirement and health reasons) for men and women who were in paid employment at age 62, by exposure (hands above shoulder height, monotonous work and high psychological demands). Adjusted for calendar year, civil status and level of education set to mean.

Results from post hoc analyses, examining the cause-specific cumulative incidence curves, show opposite associations for men and women (Figure 2). High psychological demands were associated with a decreased risk of withdrawal due to health reasons among men and an increased risk among women. Monotonous work was associated with an increased risk for both withdrawal due to retirement and health reasons among men, and a decreased risk for withdrawal due to health reasons among women. Working with hands above shoulder height was associated with a marginally increased risk of withdrawal due to retirement among men but decreased risk among women.

### Sensitivity Analyses

Sensitivity analyses on individuals born January–June 1949, having full follow-up until age 67, showed similar trends to the main analyses, although with larger uncertainties due to the smaller sample (Tables S2 and S3). Likewise, sensitivity analyses on individuals born January 1949–June 1952 showed that excluding people born after June 1952, for whom less precise employment information was available, had little effect on the results. However, using a definition of retirement solely based on flexible retirement pension, and not working hours, substantially impacted the associations between work exposures and age of retirement, particularly among women. In contrast to the main analyses, most work exposures were associated with an older age of withdrawal from paid employment for women when the operationalisation of retirement was solely based on pension drawn.

## 4. Discussion

### 4.1. Summary of the Main Findings

The study examined gender differences in associations between biomechanical and psychosocial work exposures at age 62 and age of withdrawal from paid employment. In general, the work exposures were associated with younger age of withdrawal, and the associations were stronger among men than women. The largest gender differences in the associations were found for high psychological demands, monotonous work, hands above shoulder height and high iso-strain. With the exception of high iso-strain, the gender differences were a result of the exposure only being associated with younger age of withdrawal in one gender. High psychological demands among men and monotonous work among women were associated with older age of withdrawal, and no association was observed for hands above shoulder height among women. 

### 4.2. Strengths and Limitations 

The strengths of this study included the use of high-quality longitudinal data derived from complete national registries. Data on occupation and withdrawal from paid employment were collected from different sources, and work exposure information was not influenced by time of withdrawal from paid employment. Utilising registry data also enabled us to study a large study population. 

The study also has limitations. First, causal conclusions about the observed associations cannot be made. Previous exposures and health conditions have likely affected who remains in the work force at age 62, and thus, the observed associations may be a result of selection bias induced by conditioning on being in paid employment at age 62 [32]. Furthermore, the effect of work exposure on withdrawal may differ depending on the intensity and duration of exposure and in the circumstances that led to exposure (or no exposure) at age 62. For instance, exposure at age 62 might not be representative of previous exposures, and a person may have a different withdrawal outcome depending on whether the work exposure at age 62 was long-term exposure throughout the career. Thus, a well-defined intervention may be difficult to achieve, and the underlying causal assumption of consistency is not met [33]. Additionally, if taking a causal interpretation of the results, residual confounding was likely present. For example, health prior to baseline might affect withdrawal from paid employment as well as the likelihood of being exposed to a certain work exposure.

Second, using a JEM to obtain work exposures will typically increase the risk of exposure misclassification and measurement error, as JEMs do not consider individual differences and assume homogeneity of exposure within the occupational groups [34]. The misclassification was in all probability rarer in occupational groups with gender-specific exposure estimates compared with occupational groups with combined exposure estimates, since men and women with equal occupations may have different work tasks and work exposures [35]. The performance assessment for the JEM used in this study showed fair to moderate agreement with individual exposure for all work exposures except for low leadership support, which showed poor agreement [25]. The agreement was generally better for biomechanical exposures, particularly among men. Furthermore, the JEM was developed based on a general working age population (individuals aged 18–66). Examinations of the survey data utilised when constructing the JEM showed some within-occupation dissimilarities between workers over and under 60 years. Due to this, there might be some misclassification of exposures. However, the exposure misclassification deriving from using a JEM is typically nondifferential, as the measurements of withdrawal and work exposure (occupation) are independent of each other. Consequently, the observed associations are presumably underestimated [36].

Since the exposure variables were dichotomous, the current study was not able to consider the degree of exposure (intensity, duration and frequencies). For instance, the JEM does not differentiate between working with hands raised *to* shoulder height and *high over* shoulder height, e.g., elbows over shoulder height. The latter has been found to be a stronger risk factor for the development of pain in the shoulders [37]. Accordingly, the associations between exposure and withdrawal from paid employment are presumably stronger among high-exposed than low-exposed individuals [38].

Third, as information about working hours was recorded annually, the study assumed that working hours were constant throughout the year. Since the study population consisted of older workers, any change in working hours is likely to be a decrease, which would result in an accelerated time of withdrawal. 

Fourth, changes in occupation during the follow-up period were not considered. A change in occupation after age 62 is likely to be a change to less demanding work, resulting in an overestimation of exposure and an attenuation of the associations.

Fifth, only a minority (10%) of the study population were followed until 67 years of age. However, sensitivity analyses of people born between January–June 1949, who were followed from age 62 to 67 years, showed similar results to the main analyses. 

### 4.3. Discussion and Interpretation of the Results 

In line with previous studies that reported gender-specific outcomes among older workers [14,18,20], the results from the present study indicate that biomechanical and psychosocial work exposures are associated with age of withdrawal from paid employment among both men and women and that some gender differences in the associations exist. 

The largest gender difference in the present study was observed for the association between high psychological demands and age of withdrawal. Men with low demands withdrew earlier than men with high demands, whereas opposite results were found among women. This gender difference is contrary to previous Scandinavian studies which found job stress [14] and job demands [18] to be associated with decreased risk of early retirement and disability pension in both older men and women. This discrepancy might be due to exposure and outcome heterogeneity having different effects on men and women. The present study included role conflict and emotional demands in the definition of psychological demands, and in line with the previous studies [14,18], the present study found a negative association for both genders when withdrawal from paid employment was solely based on retirement pension (i.e., working hours were excluded). This illustrates how different conceptualisations of withdrawal might lead to different results [26]. As for the present study, since it is possible to work while receiving retirement pension, it could be argued that withdrawal should be based on both working hours and pension received. The observed gender difference might be explained by a strong horizontal gender segregation in the labour market. To a large extent, men and women work in different sectors and industries with different occupations [35], and thus different job characteristics and types of demands. Alternatively, men and women may respond differently to stress, and psychosocial work exposures may impact women’s health more than men’s [39], for instance, because of women’s dual home–career workload [40].

The present study also found a gender difference in the association between monotonous work and age of withdrawal. Compared with non-exposed, exposed men withdrew earlier and exposed women withdrew slightly later. Although studies among older workers are lacking, the results are in line with Danish studies on general working age populations, suggesting that repetitive monotonous work [41] and low variation in work [42] are stronger risk factors for disability pension among men than women. Similar to psychological demands, one possible explanation for the gender difference could be that men and women have different occupations, and thus different types of monotonous work. In the present study, the most common occupation with monotonous work was stock clerks among men and shop salespersons among women, who likely have different tasks and experience monotonous work differently. 

Among the biomechanical exposures, the largest gender difference was found for working with hands above shoulder height. The association with overall working years lost was greater for men than women. This is in line with a Finnish study [43]. Cause-specific analysis showed that the gender differences mainly resulted from exposed women having a lower risk of withdrawal due to retirement compared with non-exposed women, whilst the reverse was true for men. A reason for this could be the selected study population. Individuals with the heaviest exposures or highly susceptible individuals may have left paid employment before turning 62, and the health selection might differ between men and women. 

In general, the present study found that the associations between biomechanical exposures and withdrawal from paid employment were stronger among men than women. This is contrary to previous studies. Concerning early retirement, Blekesaune and Solem [14] and d’Errico, Falkstedt, Almroth, Badarin, Hemmingsson and Kjellberg [18] both found that physical workload is associated with decreased risk among men and increased risk among women, whereas neither Krokstad, Johnsen and Westin [20] nor d’Errico, Falkstedt, Almroth, Badarin, Hemmingsson and Kjellberg [18] observed gender differences when examining the association between heavy physical demands and disability pension among older workers. Some of the discrepancies might be explained by exposure heterogeneity. For instance, in the abovementioned studies, the exposure “heavy physical demands” captures multiple biomechanical factors.

### 4.4. Generalisability

The selection process prior to employment at age 62 may affect the generalisability of the study results beyond the birth cohorts included in the study, particularly among women. For instance, the associations might change if the increase in female labour market participation results in a more diverse population of employed women.

Furthermore, the heterogeneity between countries regarding gender distribution in work participation, social systems and pension schemes can be substantial. This may limit the generalisability of the study beyond Norway. The use of a global definition of withdrawal from paid employment nevertheless strengthens the external validity, as the probability of the different work exit routes depends on social systems and current macroeconomic factors, which may vary over time and across countries. 

### 4.5. Study Implications and Future Directions

If individuals with the work exposure associated with the highest risk (standing/walking for men and low decision latitude for women) had the same mean withdrawal age as non-exposed individuals, time spent employed between age 62 and 67 would on average increase by 7% (4.4 months) among men and 5% (3.0 months) among women. However, it remains to be learned whether the increased risk of withdrawal is caused by work exposures or by certain unobserved confounding factors. 

Associations going in different directions for men and women resulted in gender differences in the risk difference (between non-exposed and exposed) ranging up to 4.4 months (7% of the time between age 62 and 67) for high psychological demands. The differences in risk factors of withdrawal among men and women indicate that workplace interventions aiming to extend working life among older workers may be more effective if occupation and gender are taken into consideration. However, more research with accurate exposure assessment is needed to better understand why men and women at age 62 have different risk factors of withdrawal from paid employment. 

## 5. Conclusions

In conclusion, the study found that work exposures at age 62 were generally associated with a younger age of withdrawal from paid employment for both genders. The associations tended to be stronger among men than women, although the gender differences were insignificant or relatively small for some work exposures. The largest gender differences were found for psychological demands (four months), monotonous work (four months) and working with hands above shoulder height (two months). For these work exposures, the findings suggest that it might be worthwhile to consider gender when implementing workplace interventions to extend working life among older workers, for instance, by targeting monotonous work typically performed by men. However, there is a strong need for further studies examining the causal pathways. The study might be biased by the selection of who remains in the workforce at age 62 and by unobserved confounding factors. 

## Figures and Tables

**Table 1:** Descriptive statistics of persons born between 1949–1953 who were in paid employment in Norway at age 62 (N = 145,331) and the proportion who withdrew from paid employment during the follow-up period 2011–2016.

	Men			Women		
	Total		Withdrew	Total		Withdrew
	N	%	%	N	%	%
**Total**	77,558	100	46	67,773	100	49
**Calendar year at baseline (birth year)**						
2011 (1949)	15,261	20	64	13,183	20	68
2012 (1950)	14,866	19	56	13,032	19	62
2013 (1951)	15,202	19	46	13,074	19	50
2014 (1952)	16,040	21	37	14,003	21	39
2015 (1953)	16,189	21	29	14,481	21	29
**Civil status**						
Single	20,457	26	48	22,280	33	44
Married/Cohabiting	56,962	73	45	45,442	67	52
Unknown	139	<1	60	51	<1	51
**Education level**						
Lower secondary or lower	9460	12	54	8186	12	54
Upper secondary basic level	18,584	24	51	22,565	33	52
Upper secondary final year	21,093	27	49	12,413	18	48
Bachelor	18,051	24	42	20,522	30	48
Master or higher	10,102	13	30	3951	6	31
Unknown	268	<1	51	136	<1	49
**Biomechanical exposures ^1^**						
Heavy lifting	34,035	44	52	18,717	28	54
Neck flexion	12,718	16	53	18,736	28	52
Hands above shoulder height	19,485	25	52	10,164	15	52
Squatting/kneeling	10,216	13	54	15,630	23	55
Forward bending	6052	8	53	9174	14	52
Awkward lifting	7079	9	55	13,693	20	54
Heavy physical work	24,254	31	53	15,631	23	54
Standing/walking	30,764	40	53	39,001	58	52
**Psychosocial exposures ^1^**						
Monotonous work	32,150	41	52	29,717	44	50
Low supportive leadership	40,110	52	47	40,894	60	49
High psychological demands	37,130	48	40	35,883	53	48
High emotional demands	23,711	31	46	50,887	75	49
Low decision latitude	25,976	33	53	49,727	73	51
Job strain	7256	9	48	24,328	36	51
Iso-strain	6041	8	48	23,468	35	51

^1^ The exposure categories are not mutually exclusive.

**Table 2:** Distribution of cause-specific withdrawal among persons born between 1949–1953 who were in paid employment in Norway at age 62 and who withdrew from paid employment during the follow-up period 2011–2016 (N = 68,967).

	Men		Women	
Withdrawal from Paid Employment	N	%	N	%
All-cause withdrawal	35,609	100	33,358	100
Withdrawal due to retirement	22,202	62	18,814	56
Withdrawal due to health reasons	8782	25	10,073	30
Withdrawal due to unemployment	1256	4	615	2
Withdrawal due to other reasons	2889	8	3648	11
Withdrawal due to death or emigration	480	1	208	1

**Table 3:** Difference in mean estimated time (months) until all-cause withdrawal from paid employment between non-exposed and exposed workers in paid employment at age 62, and the gender difference in this difference.

	Men		Women		Gender Difference
	Crude ^1^ (95% CI ^2^)	Adjusted ^3^ (95% CI)	Crude ^1^ (95% CI)	Adjusted ^3^ (95% CI)	Adjusted ^4^ (95% CI)
**Biomechanical exposures**					
Heavy lifting	5.90 (5.68–6.41)	3.02 (2.63–3.42)	3.29 (3.02–3.58)	2.74 (2.33–3.14)	0.28 (−0.29–0.83)
Neck flexion	4.13 (3.90–4.59)	1.97 (1.50–2.43)	2.27 (1.94–2.43)	1.87 (1.47–2.29)	0.09 (−0.53–0.71)
Hands above shoulder height	4.35 (4.00–4.57)	2.33 (1.94–2.74)	1.16 (0.76–1.87)	−0.08 (−0.62–0.47)	2.41 (1.76–3.10)
Squatting/kneeling	5.17 (4.89–5.62)	2.68 (2.19–3.20)	3.66 (3.37–3.84)	2.91 (2.49–3.36)	−0.23 (−0.87–0.44)
Forward bending	3.94 (3.61–4.32)	1.85 (1.23–2.47)	1.86 (1.67–2.29)	1.34 (0.82–1.84)	0.51 (−0.25–1.33)
Awkward lifting	5.28 (4.92–5.65)	2.69 (2.08–3.24)	2.89 (2.41–3.21)	2.28 (1.85–2.74)	0.41 (−0.34–1.13)
Heavy physical work	5.70 (5.44–5.94)	2.98 (2.59–3.39)	3.02 (2.98–3.55)	2.33 (1.89–2.83)	0.65 (0.03–1.26)
Standing/walking	6.24 (5.97–6.47)	4.43 (4.07–4.81)	3.73 (3.40–3.97)	2.95 (2.57–3.32)	1.48 (0.98–2.00)
**Psychosocial exposures**					
Monotonous work	5.38 (5.18–5.58)	2.83 (2.45–3.19)	0.19 (−0.07–0.39)	−1.29 (−1.68–−0.87)	4.12 (3.51–4.69)
Low supportive leadership	1.09 (0.86–1.17)	1.02 (0.67–1.37)	0.55 (0.31–0.82)	1.18 (0.80–1.53)	−0.16 (−0.68–0.36)
High psychological demands	−6.01 (−6.38–−5.69)	−3.66 (−4.04–−3.25)	−0.78 (−0.99–−0.63)	0.71 (0.28–1.10)	−4.37 (−4.97–−3.81)
Emotional demands	−0.08 (−0.35–0.28)	1.84 (1.48–2.24)	−0.33 (−0.67–0.03)	0.13 (−0.26–0.59)	1.71 (1.17–2.29)
Low decision latitude	5.54 (5.12–5.85)	4.07 (3.70–4.45)	4.17 (3.92–4.52)	3.00 (2.58–3.43)	1.07 (0.49–1.63)
High job strain	1.89 (1.26–2.67)	3.89 (3.30–4.50)	1.71 (1.34–1.98)	2.06 (1.64–2.44)	1.83 (1.13–2.60)
High iso-strain	1.41 (0.87–1.84)	4.14 (3.47–4.84)	1.68 (1.37–1.83)	2.00 (1.61–2.40)	2.14 (1.38–2.95)

^1^ Employment time_non-exposed_ − Employment time_exposed_. ^2^ 95% CIs based on 1000 bootstrap samples. ^3^ As ^1^ but adjusted for calendar year, civil status and level of education set to mean. ^4^ (Employment time_non-exposed men_ − Employment time_exposed men_) − (Employment time_non-exposed women_ − Employment time _exposed women_), adjusted for calendar year, civil status and level of education set to mean.

## Data Availability

Restrictions apply to the availability of these data, which were used under license for the current study, and so are not publicly available. Data supporting reported results are available from Statistics Norway.

## Supplementary Materials

The following supporting information can be downloaded at: https://www.mdpi.com/article/10.3390/ijerph191710563/s1, Table S1: Gender-specific Cox proportional hazard ratio of all-cause withdrawal from paid employment according to work exposure; Table S2: Cox proportional hazard ratio of all-cause withdrawal from paid employment according to work exposure among men, main analysis and sensitivity analyses; Table S3: Cox proportional hazard ratio of all-cause withdrawal from paid employment according to work exposure among women, main analysis and sensitivity analyses; Figure S1: Difference between non-exposed and exposed in probability of being in paid employment at age 62–67 (i.e., difference in survival curves), biomechanical exposures; Figure S2: Difference between non-exposed and exposed in probability of being in paid employment at age 62–67 (i.e., difference in survival curves), psychosocial exposures.

## References

[1] OECD (2017). Pensions at a Glance 2017: OECD and G20 Indicators.

[2] Dahl E., Lien O.C. (2013). Pensjonsreformen–flere eldre i arbeid [The pension reform—more elderly people at work]. Arb. Og Velferd.

[3] Nilsson K. (2020). A sustainable working life for all ages–The swAge-model. Appl. Ergon..

[4] Andersen L.L., Jensen P.H., Sundstrup E. (2020). Barriers and opportunities for prolonging working life across different occupational groups: The SeniorWorkingLife study. Eur. J. Public Health.

[5] Reeuwijk K.G., De Wind A., Westerman M.J., Ybema J.F., Van der Beek A.J., Geuskens G.A. (2013). ‘All those things together made me retire’: Qualitative study on early retirement among Dutch employees. BMC Public Health.

[6] de Wind A., Geuskens G.A., Ybema J.F., Bongers P.M., van der Beek A.J. (2015). The role of ability, motivation, and opportunity to work in the transition from work to early retirement–testing and optimizing the Early Retirement Model. Scand. J. Work Environ. Health.

[7] Armstrong-Stassen M., Ursel N.D. (2009). Perceived organizational support, career satisfaction, and the retention of older workers. J. Occup. Organ. Psychol..

[8] Robroek S.J., Schuring M., Croezen S., Stattin M., Burdorf A. (2013). Poor health, unhealthy behaviors, and unfavorable work characteristics influence pathways of exit from paid employment among older workers in Europe: A four year follow-up study. Scand. J. Work Environ. Health.

[9] Thorsen S.V., Jensen P.H., Bjørner J.B. (2016). Psychosocial work environment and retirement age: A prospective study of 1876 senior employees. Int. Arch. Occup. Environ. Health.

[10] Lund T., Villadsen E. (2005). Who retires early and why? Determinants of early retirement pension among Danish employees 57–62 years. Eur. J. Ageing.

[11] Lund T., Iversen L., Poulsen K.B. (2001). Work environment factors, health, lifestyle and marital status as predictors of job change and early retirement in physically heavy occupations. Am. J. Ind. Med..

[12] De Wind A., Burr H., Pohrt A., Hasselhorn H.M., Van der Beek A.J., Rugulies R. (2017). The association of health and voluntary early retirement pension and the modifying effect of quality of supervision: Results from a Danish register-based follow-up study. Scand. J. Public Health.

[13] De Wind A., Geuskens G.A., Ybema J.F., Blatter B.M., Burdorf A., Bongers P.M., Van der Beek A.J. (2014). Health, job characteristics, skills, and social and financial factors in relation to early retirement-results from a longitudinal study in the Netherlands. Scand. J. Work Environ. Health.

[14] Blekesaune M., Solem P.E. (2005). Working conditions and early retirement: A prospective study of retirement behavior. Res. Aging.

[15] Friis K., Ekholm O., Hundrup Y.A., Obel E.B., Grønbæk M. (2007). Influence of health, lifestyle, working conditions, and sociodemography on early retirement among nurses: The Danish Nurse Cohort Study. Scand. J. Public Health.

[16] Sundstrup E., Hansen Å.M., Mortensen E.L., Poulsen O.M., Clausen T., Rugulies R., Møller A., Andersen L.L. (2018). Retrospectively assessed physical work environment during working life and risk of sickness absence and labour market exit among older workers. Occup. Environ. Med..

[17] Sundstrup E., Thorsen S.V., Rugulies R., Larsen M., Thomassen K., Andersen L.L. (2021). Importance of the Working Environment for Early Retirement: Prospective Cohort Study with Register Follow-Up. Int. J. Environ. Res. Public Health.

[18] D’Errico A., Falkstedt D., Almroth M., Badarin K., Hemmingsson T., Kjellberg K. (2022). Long-term sick leave for back pain, exposure to physical workload and psychosocial factors at work, and risk of disability and early-age retirement among aged Swedish workers. Int. Arch. Occup. Environ. Health.

[19] D’Errico A., Burr H., Pattloch D., Kersten N., Rose U. (2020). Working conditions as risk factors for early exit from work—In a cohort of 2351 employees in Germany. Int. Arch. Occup. Environ. Health.

[20] Krokstad S., Johnsen R., Westin S. (2002). Social determinants of disability pension: A 10-year follow-up of 62 000 people in a Norwegian county population. Int. J. Epidemiol..

[21] Soidre T. (2005). Retirement-age preferences of women and men aged 55–64 years in Sweden. Ageing Soc..

[22] Claussen B., Dalgard O.S. (2009). Disability pensioning: The gender divide can be explained by occupation, income, mental distress and health. Scand. J. Public Health.

[23] NAV Statistikknotat (2021). Utviklingen i Alderspensjon per 30. juni 2021 [Developments in Retirement Pensions as of 30 June 2021].

[24] Statistics Norway Variable Lists. https://www.ssb.no/data-til-forskning/utlan-av-data-til-forskere/variabellister.

[25] Hanvold T.N., Sterud T., Kristensen P., Mehlum I.S. (2019). Mechanical and psychosocial work exposures: The construction and evaluation of a gender-specific job exposure matrix (JEM). Scand. J. Work Environ. Health.

[26] Leinonen T., Boets I., Pletea E., Vandenbroeck S., Sivesind Mehlum I., Hasselhorn H.M., de Wind A. (2022). A conceptual framework addressing the complex labour market dynamics of the work-to-retirement process. Eur. J. Ageing.

[27] Jacobsen O. (2014). Pensjonsreformen: Hvilken innvirkning har den hatt på bruken av helserelaterte ytelser [The pension reform: What impact has it had on the use of health-related benefits]. Arb. Og Velferd.

[28] Statistics Norway Standard for Yrkesklassifisering (Standard Classification of Occupations). https://www.ssb.no/a/publikasjoner/pdf/nos_c521/nos_c521.pdf.

[29] Lindström K. (2000). User’s Guide for the QPSNordic: General Nordic Questionnaire for Psychological and Social Factors at Work.

[30] Statistics Norway Norwegian Standard Classification of Education. https://www.ssb.no/en/utdanning/norwegian-standard-classification-of-education.

[31] Andersen P.K., Geskus R.B., de Witte T., Putter H. (2012). Competing risks in epidemiology: Possibilities and pitfalls. Int. J. Epidemiol..

[32] Cole S.R., Platt R.W., Schisterman E.F., Chu H., Westreich D., Richardson D., Poole C. (2009). Illustrating bias due to conditioning on a collider. Int. J. Epidemiol..

[33] Hernán M., Robins J. (2020). Causal Inference: What If.

[34] Peters S. (2020). Although a valuable method in occupational epidemiology, job-exposure -matrices are no magic fix. Scand. J. Work Environ. Health.

[35] Ose S.O., Jiang L., Bungum B. (2014). Det Kjønnsdelte Arbeidsmarkedet og Kvinners Arbeidshelse [The Gender-Segregated Labor Market and Women’s Occupational Health].

[36] Armstrong B.G. (1998). Effect of measurement error on epidemiological studies of environmental and occupational exposures. Occup. Environ. Med..

[37] Wærsted M., Koch M., Veiersted K.B. (2020). Work above shoulder level and shoulder complaints: A systematic review. Int. Arch. Occup. Environ. Health.

[38] Jansen J.P., Morgenstern H., Burdorf A. (2004). Dose-response relations between occupational exposures to physical and psychosocial factors and the risk of low back pain. Occup. Environ. Med..

[39] Herrero S.G., Saldaña M.Á.M., Rodriguez J.G., Ritzel D.O. (2012). Influence of task demands on occupational stress: Gender differences. J. Saf. Res..

[40] Lundberg U., Frankenhaeuser M. (1999). Stress and workload of men and women in high-ranking positions. J. Occup. Health Psychol..

[41] Albertsen K., Lund T., Christensen K.B., Kristensen T.S., Villadsen E. (2007). Predictors of disability pension over a 10-year period for men and women. Scand. J. Public Health.

[42] Christensen K.B., Feveile H., Labriola M., Lund T. (2008). The impact of psychosocial work environment factors on the risk of disability pension in Denmark. Eur. J. Public Health.

[43] Schram J.L., Solovieva S., Leinonen T., Viikari-Juntura E., Burdorf A., Robroek S.J. (2020). The influence of occupational class and physical workload on working life expectancy among older employees. Scand. J. Work Environ. Health.

