# Supplementary material for: Gender Differences in Associations between Biomechanical and Psychosocial Work Exposures and Age of Withdrawal from Paid Employment among Older Workers
Source: Int J Environ Res Public Health. 2022 Aug 24;19(17):10563. doi: 10.3390/ijerph191710563 (PMC9518318; doi:10.3390/ijerph191710563)
Supplement: Supplementary file 1 [file ijerph-19-10563-s001.zip › ijerph-1818878-supplementary.pdf]

International Journal of
Environmental Research and Public Health

Supplementary Materials:

Table S1: Gender-specific Cox proportional hazard ratio of all-cause withdrawal from paid employment according to work exposure

	Men		Women	
	Crude	*Adjusted*[1]	*Crude*	*Adjusted*[1]
	HR (95% CI)	HR (95% CI)	HR (95% CI)	HR (95% CI)
**Biomechanical exposures**				
Heavy lifting	1.43 (1.40–1.46)	1.20 (1.17–1.23)	1.21 (1.18–1.24)	1.17 (1.15–1.20)
Neck flexion	1.28 (1.24–1.31)	1.13 (1.09–1.16)	1.14 (1.11–1.17)	1.12 (1.09–1.14)
Hands above shoulder height	1.29 (1.26–1.32)	1.15 (1.12–1.18)	1.08 (1.05–1.11)	1.00 (0.96–1.03)
Squatting/kneeling	1.35 (1.31–1.39)	1.17 (1.14–1.21)	1.24 (1.21–1.27)	1.19 (1.16–1.22)
Forward bending	1.26 (1.22–1.31)	1.12 (1.08–1.16)	1.11 (1.08–1.15)	1.08 (1.05–1.12)
Awkward lifting	1.36 (1.32–1.41)	1.17 (1.13–1.21)	1.18 (1.15–1.21)	1.14 (1.11–1.17)
Heavy physical work	1.40 (1.37–1.43)	1.20 (1.17–1.23)	1.19 (1.17–1.22)	1.15 (1.12–1.18)
Standing/walking	1.46 (1.43–1.49)	1.31 (1.28–1.34)	1.25 (1.22–1.27)	1.19 (1.17–1.22)
**Psychosocial exposures**				
Monotonous work	1.38 (1.36–1.41)	1.19 (1.16–1.21)	1.02 (1.00–1.04)	0.93 (0.90–0.95)
Low supportive leadership	1.07 (1.05–1.09)	1.06 (1.04–1.09)	1.03 (1.01–1.05)	1.07 (1.05–1.10)
High psychological demands	0.69 (0.68–0.71)	0.80 (0.78–0.82)	0.95 (0.93–0.97)	1.04 (1.02–1.07)
Emotional demands	1.00 (0.97–1.02)	1.12 (1.09–1.14)	0.98 (0.95–1.00)	1.01 (0.98–1.03)
Low decision latitude	1.39 (1.36–1.42)	1.28 (1.25–1.31)	1.29 (1.26–1.32)	1.20 (1.17–1.23)
High job strain	1.12 (1.08–1.16)	1.26 (1.21–1.31)	1.10 (1.08–1.13)	1.13 (1.10–1.16)
High iso-strain	1.09 (1.05–1.13)	1.28 (1.23–1.33)	1.10 (1.07–1.12)	1.13 (1.10–1.15)

[1] Adjusted for civil status, level of education and calendar year at baseline

Table S2: Cox proportional hazard ratio of all-cause withdrawal from paid employment according to work exposure among men, main analysis and sensitivity analyses

	Men			
	Main analysis (N=77,558)	*Full follow up until age 67 (born January - June 1949)*[1] *(N=8,100)*	*More precise employment information (born 1949 - June 1952)*[2] *(N= 53,528)*	*Different definition of early retirement*[2,3]
	HR (95% CI)	*HR (95% CI)*	*HR (95% CI)*	*HR (95% CI)*
**Biomechanical exposures**				
Heavy lifting	1.20 (1.17–1.23)	1.15 (1.08–1.23)	1.20 (1.17–1.24)	1.11 (1.09–1.13)
Neck flexion	1.13 (1.09–1.16)	1.11 (1.03–1.20)	1.13 (1.10–1.17)	1.13 (1.11–1.15)
Hands above shoulder height	1.15 (1.12–1.18)	1.11 (1.04–1.19)	1.15 (1.12–1.18)	1.06 (1.04–1.08)
Squatting/kneeling	1.17 (1.14–1.21)	1.13 (1.04–1.23)	1.18 (1.14–1.22)	1.11 (1.08–1.13)
Forward bending	1.12 (1.08–1.16)	1.08 (0.97–1.19)	1.12 (1.08–1.17)	1.11 (1.08–1.15)
Awkward lifting	1.17 (1.13–1.21)	1.09 (0.99–1.20)	1.17 (1.13–1.22)	1.13 (1.10–1.16)
Heavy physical work	1.20 (1.17–1.23)	1.15 (1.08–1.23)	1.20 (1.17–1.24)	1.08 (1.06–1.10)
Standing/walking	1.31 (1.28–1.34)	1.30 (1.23–1.38)	1.32 (1.29–1.35)	1.05 (1.03–1.06)
**Psychosocial exposures**				
Monotonous work	1.19 (1.16–1.21)	1.19 (1.12–1.26)	1.19 (1.16–1.22)	1.16 (1.14–1.18)

Low supportive leadership	1.06 (1.04–1.09)	1.04 (0.98–1.09)	1.06 (1.03–1.08)	0.97 (0.95–0.98)
High psychological demands	0.80 (0.78–0.82)	0.88 (0.83–0.93)	0.81 (0.79–0.83)	0.85 (0.84–0.87)
Emotional demands	1.12 (1.09–1.14)	1.18 (1.11–1.26)	1.13 (1.10–1.16)	0.95 (0.93–0.97)
Low decision latitude	1.28 (1.25–1.31)	1.31 (1.24–1.39)	1.28 (1.24–1.31)	1.08 (1.06–1.10)
Job strain	1.26 (1.21–1.31)	1.42 (1.30–1.56)	1.29 (1.24–1.34)	0.86 (0.83–0.88)
Iso-strain	1.28 (1.23–1.33)	1.39 (1.25–1.54)	1.30 (1.25–1.36)	0.88 (0.85–0.91)

[1]Adjusted for civil status and educational level

[2]Adjusted for calendar year at baseline, civil status and educational level

[3]Definition of early retirement is "receiving at least 50% flexible retirement pension" (working hours are not included in the definition).

Table S3: Cox proportional hazard ratio of all-cause withdrawal from paid employment according to work exposure among women, main analysis and sensitivity analyses

	**Women**			
	Main analyses (N=67,773)	*Full follow up until age 67 (born January - June 1949)[1] (N=6,895)*	*More precise employment information (born 1949 - June 1952)[2] (N=46,511)*	*Different definition of early retirement[2, 3]*
	HR (95% CI)	*HR (95% CI)*	*HR (95% CI)*	*HR (95% CI)*
**Biomechanical exposures**				
Heavy lifting	1.17 (1.15–1.20)	1.10 (1.03–1.17)	1.16 (1.13–1.19)	0.98 (0.96–1.00)
Neck flexion	1.12 (1.09–1.14)	1.12 (1.06–1.20)	1.13 (1.10–1.16)	0.89 (0.87–0.91)
Hands above shoulder height	1.00 (0.96–1.03)	1.01 (0.93–1.10)	1.00 (0.97–1.04)	0.87 (0.85–0.90)
Squatting/kneeling	1.19 (1.16–1.22)	1.12 (1.05–1.21)	1.19 (1.16–1.23)	0.81 (0.79–0.83)
Forward bending	1.08 (1.05–1.12)	1.02 (0.94–1.11)	1.09 (1.05–1.12)	0.84 (0.81–0.86)
Awkward lifting	1.14 (1.11–1.17)	1.08 (1.01–1.17)	1.13 (1.10–1.17)	0.88 (0.86–0.90)
Heavy physical work	1.15 (1.12–1.18)	1.13 (1.05–1.21)	1.14 (1.11–1.18)	0.85 (0.83–0.87)
Standing/walking	1.19 (1.17–1.22)	1.15 (1.08–1.22)	1.19 (1.16–1.22)	0.81 (0.79–0.82)
**Psychosocial exposures**				
Monotonous work	0.93 (0.90–0.95)	0.95 (0.89–1.01)	0.93 (0.90–0.95)	1.08 (1.06–1.10)
Low supportive leadership	1.07 (1.05–1.10)	1.11 (1.04–1.17)	1.07 (1.05–1.10)	0.90 (0.89–0.92)
High psychological demands	1.04 (1.02–1.07)	1.06 (1.00–1.13)	1.05 (1.02–1.08)	0.91 (0.89–0.93)
Emotional demands	1.01 (0.98–1.03)	1.05 (0.98–1.12)	1.01 (0.98–1.04)	0.84 (0.82–0.86)
Low decision latitude	1.20 (1.17–1.23)	1.15 (1.07–1.23)	1.18 (1.15–1.22)	0.92 (0.90–0.94)
Job strain	1.13 (1.10–1.16)	1.10 (1.04–1.18)	1.13 (1.10–1.16)	0.89 (0.87–0.91)
Iso-strain	1.13 (1.10–1.15)	1.11 (1.05–1.19)	1.13 (1.10–1.16)	0.87 (0.86–0.89)

[1]Adjusted for civil status and educational level

[2]Adjusted for calendar year at baseline, civil status and educational level

[3]Definition of early retirement is "receiving at least 50% flexible retirement pension" (working hours are not included in the definition).

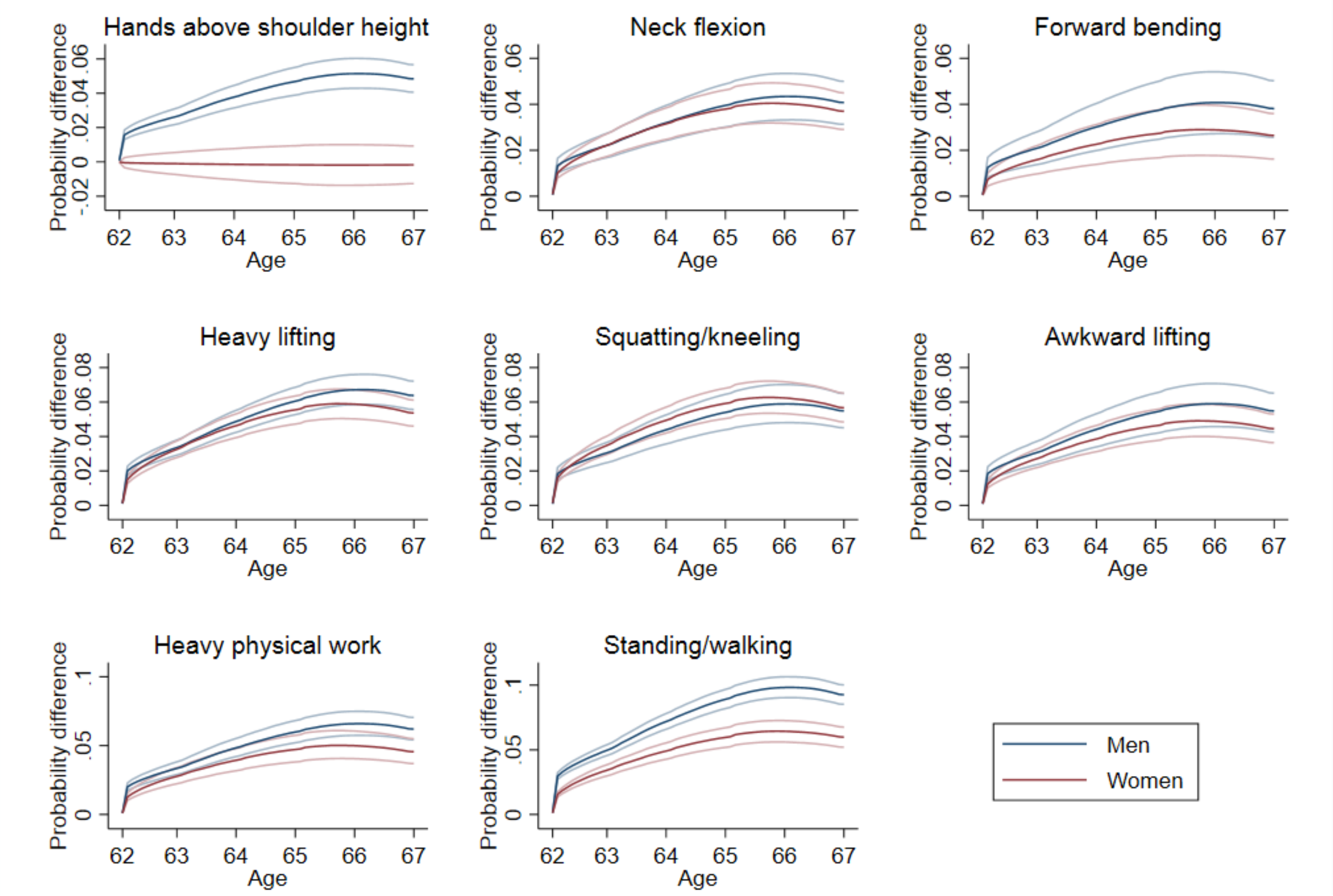

Figure S1: Difference between non-exposed and exposed in probability of being in paid employment at age 62 - 67 (i.e., difference in survival curves), biomechanical exposures. Note that Y axis scale varies between graphs

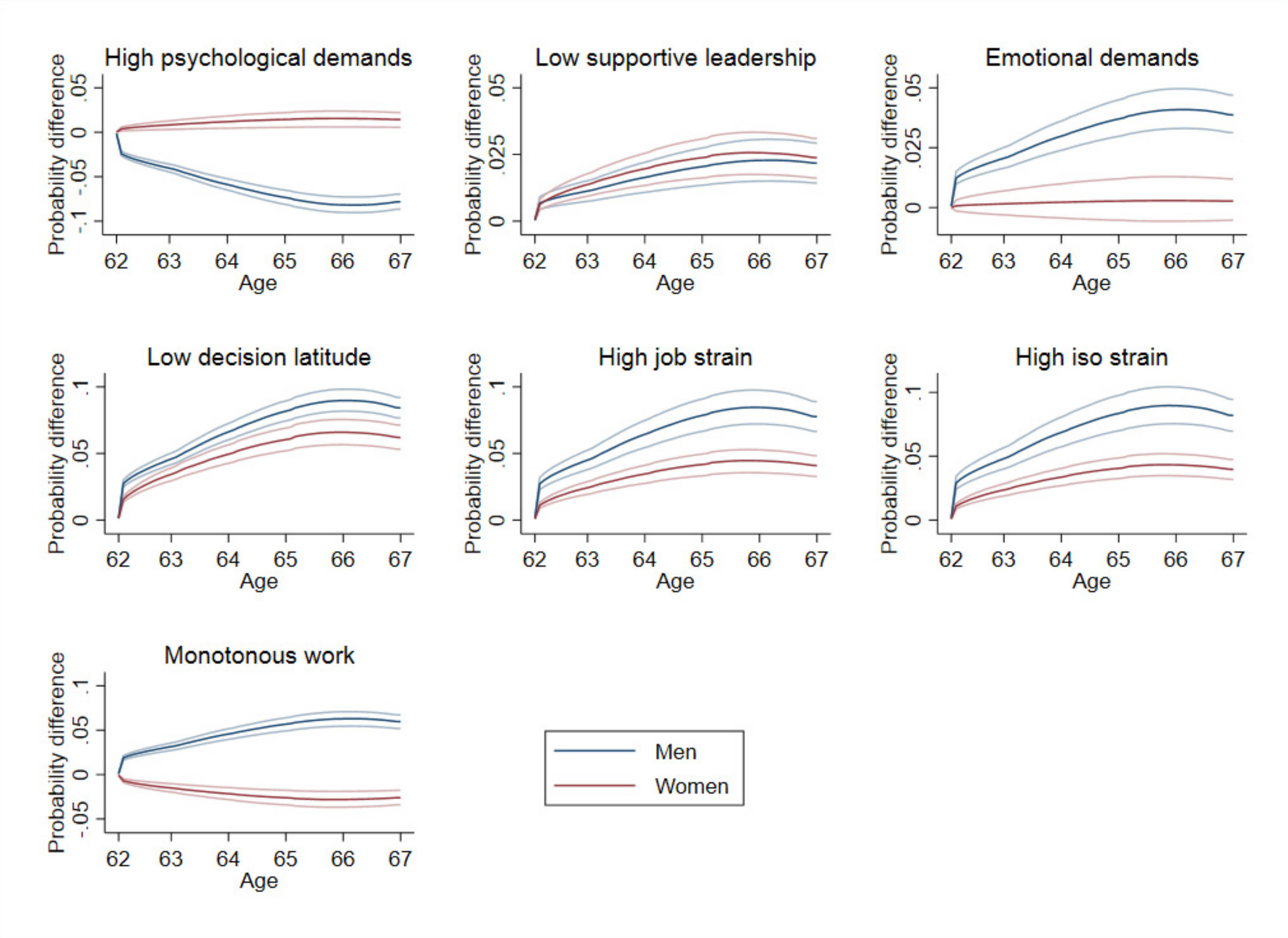

Figure S2: Difference between non-exposed and exposed in probability of being in paid employment at age 62 - 67 (i.e., difference in survival curves), psychosocial exposures. Note that Y axis scale varies between graphs
